# Circulating Serum miRNAs as Diagnostic Markers for Colorectal Cancer

**DOI:** 10.1371/journal.pone.0154130

**Published:** 2016-05-02

**Authors:** Abdel-Rahman N. Zekri, Amira Salah El-Din Youssef, Mai M. Lotfy, Reham Gabr, Ola S. Ahmed, Auhood Nassar, Nehal Hussein, Dalia Omran, Eman Medhat, Salam Eid, Marwa Mahmoud Hussein, Maha Yahia Ismail, Faris Q. Alenzi, Abeer A. Bahnassy

**Affiliations:** 1 Molecular Virology and Immunology Unit, Cancer Biology Department, National Cancer Institute, Cairo University, Cairo, Egypt; 2 Tropical Medicine Department, Kasr El- Aini hospital, Faculty of Medicine, Cairo University, Cairo, Egypt; 3 Medical Oncology Department, National Cancer Institute, Cairo University, Cairo, Egypt; 4 Department of Clinical Laboratory Sciences, College of Applied Medical Sciences, King Saud University, Alkharaj, Saudi Arabia; 5 Pathology Department, National Cancer Institute, Cairo University, Cairo, Egypt; University of Kansas School of Medicine, UNITED STATES

## Abstract

**Aim:**

The study was designed to assess the possibility of using circulating miRNAs (serum miRNAs) as diagnostic biomarkers in colorectal cancer (CRC) and to identify their possibility as candidates for targeted therapy.

**Methods:**

The study involved two sample sets: 1- a training set which included 90 patients with colorectal related disease (30 with CRC, 18 with inflammatory bowel disease (IBD), 18 with colonic polyps (CP) and 24 with different colonic symptoms but without any colonoscopic abnormality who were enrolled as control group) and 2- a validation set which included 100 CRC patients. Serum miRNAs were extracted from all subjects to assess the expression profiles for the following miRNAs (*miR-17*, *miR-18a*, *miR-19a*, *miR-19b*, *miR-20a*, *miR-21*, *miR-146a*, *miR-223*, *miR-24*, *miR-454*, *miR-183*, *miR-135a*, *miR- 135b and miR- 92a*) using the custom miScript miRNA PCR-based sybergreen array. The area under the receiver operating characteristic curve (AUC) was used to evaluate the diagnostic performance of the studied miRNAs for colorectal cancer diagnosis.

**Results:**

Data analysis of miRNA from the training set showed that; compared to control group, only ***miR-19b*** was significantly up-regulated in patients with IBD group (fold change = 5.24, p = 0.016), whereas in patients with colonic polyps, ***miR-18a*** was significantly up-regulated (fold change = 3.49, p-value = 0.018). On the other hand, *miR-17*, *miR-19a*, *miR-20a and* ***miR-223*** were significantly up-regulated (fold change = 2.35, 3.07, 2.38 and 10.35; respectively and p-value = 0.02, 0.015, 0.017 and 0.016; respectively in CRC patients. However, the validation set showed that only ***miR-223*** was significantly up-regulated in CRC patients (fold change = 4.06, p-value = 0.04).

**Conclusion:**

Aberrant miRNA expressions are highly involved in the cascade of colorectal carcinogenesis. We have found that (*miR-17*, *miR-19a*, *miR-20a and miR-223*) could be used as diagnostic biomarkers for CRC. On the other hand, *miR-19b* and *miR-18a* could be used as diagnostic biomarkers for CP and IBD respectively.

## Introduction

Colorectal cancer (CRC) is one of the most common malignant neoplasms worldwide, being the second in females and the third in males with 1.2 million annual new cases worldwide [1]. Despite the increased awareness as well as improved screening recommendations and techniques, CRC remains the second leading cause of cancer-related death in males and females [2] and being responsible for 10% of the cancer- related mortality world-wide [3].

It has been previously addressed that patients with inflammatory bowel disease (IBD) are usually associated with an increased risk of progression to epithelial dysplasia and CRC [4, 5].

The miRNAs represent an interesting class of small (18–25 nucleotides long) noncoding RNAs that act as posttranscriptional regulators of gene expression through binding to the 3`untranslated regions (UTR) of the target mRNAs and promoting mRNA degradation or translational repression [6]. The fact that miRNAs play a role in cancer biology was supported by finding that more than 50% of the miRNA genes are located at the fragile sites and regions of deletion or amplifications, which are altered in different types of human cancer [7]. Cumulative evidence indicates that some miRNAs can behave either as oncomirs or tumor suppressor genes in the cascade of CRC.Therefore, they possess the potentiality to be used as diagnostic, prognostic or therapeutic tumor markers [8].

Different studies have reported significant changes of miRNA expression levels in CRC tissues compared to normal colonic epithelium, and identified groups of miRNAs that enable prognostic stratification of CRC patients [9]. In this context, increased expression of many miRNAs, which mediate cell growth and tumor progression, was reported in the blood and/or tissues of CRC cases using a miRNA microarray assay that *included miR-20*, *miR-21*, *miR-17-5p*, *miR-15b*, *miR-181b*, *miR-191 and miR-200c* [10, 11]. The presence of miRNAs in serum, plasma and other body fluids such as urine, saliva, and amniotic fluid encouraged miRNAs research since this facilitates their detection and makes them ideal candidates as non-invasive biomarkers for early detection and monitoring disease progression [12].

The aim of the current study is to assess the role of aberrant miRNAs expressions in the development and progression of CRC cases. This was accomplished through studying the expression levels of 14 miRNAs at different stages of colorectal carcinogenesis cascade. The rational for selection of studied 14 miRNAs was based on prior references which illustrated their role in colorectal carcinogenesis **(S1 Table).**

## Patients and Methods

### Study Design

Two independent sample sets were included in this study (the training set and the validation set). The training set included 90 patients who attended the gastrointestinal endoscopy unit of the tropical medicine department, Kasr El-Aini School of medicine, Cairo University; during the period from January 2011 to March 2012. Based on colonoscopic results and histopathological examination of the studied cases, patients were classified into four groups; 30 patients with CRC, 18 with inflammatory bowel disease (IBD), 18 with colonic polyps (CP) and 24 with different colonic symptoms but without any colonoscopic abnormality who served as a control group. The validation set included 100 CRC cases, which were diagnosed and treated the National Cancer Institute, Cairo University during the period from January 2013 to March 2014. A written informed consent was obtained from each patient after the approval of the ethical committees of the NCI (National Cancer Institute), Cairo University. The IRB members are Prof. Ahmed Morsi Mostafa, Vice Dean for Higher Education and Research; Prof.Wahid Yousry, Head of Surgical Oncology Dept.; Prof. Emad El-Gemaei, Head of Pathology Dept.; Prof. Rabab Gaafar, Head of Medical Oncology Dept.; Prof. Mahmoud El Gebaly, Head of Clincal Pathology Dept.; Prof. Mervat El Nagar, Head of Radiotherapy Dept.; Prof. Sabry Shaarawy, Head of Cancer Biology Dept.; Prof. Ikram Hamed, Head of Radiodiagnosis Dept.; Prof. Mai Helali, Head of Anathesia and Pain control Dept.; Prof. Nelly Hassan, Head of Cancer Epidemiology and Biostatistics Dept.and Dr. Atef Badran, Senior of Clinical Data Manager. Organization No.IORG0003381. IRB NO.IRB00004025.

### Serum samples collection

From each patient and control subject, 5ml of venous blood were obtained, centrifuged and the serum was separated. All serum samples were stored at -80°C until used.

### RNA extraction and qRT-PCR

Total RNA including miRNAs was extracted from sera using the miRNeasy Mini Kit (Qiagen, Germany, Cat. No.217004). According to the manufacturer's instructions, c-DNA Synthesis was performed using miScript II RT kit (Qiagen, Cat. No.218161). Quantitative real-time PCR (qRT-PCR) was performed using miScript syber green PCR kit (Qiagen, Cat. No.218075). Gene expression profiles were generated in 96-well arrays using the custom miScript miRNA PCR array for the following microRNAs (***miR-17*, *miR-18a*, *miR-19a*, *miR-19b*, *miR-20a*, *miR-21*, *miR-146a*, *miR-223*, *miR-24*, *miR-454*, *miR-183*, *miR- 135a*, *miR- 135b and miR- 92a****)* (Qiagen, Cat. No. CM1HS0064C) according to manufacturers' instruction as follows: 15 min at 95°C for 1 cycle, 15s at 94°C, 30s at 55°C, and 30s at 70°C for 40 cycles using AB 7500 Fast Real-Time PCR system. Threshold cycle data were analyzed using the RT2 Profiler software (version 3.4; SABiosciences). Relative gene expression levels were normalized to housekeeping gene (SNORD 68) and the fold changes of the target gene(s) expression relative to those of the control group were analyzed by the 2−ΔΔCT method.

### Statistical analysis

The relative expression of miRNAs was analyzed using SABiosciences software and the p-value was calculated based on a Student's t-test of the replicate 2^ (- Delta Ct) values for each gene in the control and treatment groups. P-value less than 0.05 was considered significant. Receiver-operating-characteristic (ROC) curves and the area under the curve (AUC) were used to assess the diagnostic accuracy of serum miRNAs. The relevant clinicopathological features were determined by one way ANOVA for quantitative data and X^2^ test for qualitative data. All tests were 2-sided and p-value <0.05 was considered statistically significant. Statistical analysis was relied on SPSS 17.0 software (SPSS Ltd., UK) and graphs were generated with Graph pad Prism 5.0 (Graph pad Software Inc, USA).

## Results

### Clinical features of the studied groups

Regarding the training set, the mean age of the CRC patients (51.10± 10) was significantly higher than other groups **(p-value<0.05)**. However, no significant difference was reported between the mean age of the control group (43.7 ±14.2) and those with CP (40.00 ± 15.0) and IBD (41.6 ± 15.4) or the sex for all groups **(p-value >0.48)**. Additionally, there were statistically significant differences between the studied groups in relation to abdominal pain, mucus, loss of weight, and anemia **(p-value <0.05)**. Regarding the validation set, there was no significant difference between the mean age of CRC group (46.7± 14.5) and the control group (43.7 ±14.2) or the sex between the two groups. However, there were statistically significant differences between the CRC group and the control group in relation to abdominal pain, mucus, loss of weight, and anemia **(p-value <0.05)** as shown in **(Table 1)**.

**Table 1:** The clinical data of the studied groups.

Parameters	Control(n = 24)	IBD (n = 18)	Colonic polyps (n = 18)	CRC (n = 30)Training set	CRCa (n = 100) validation set	P-value
**Gender n (%)**Male	15 (62.5%) ^**a**^	15 (83.3%) ^**a**^	12 (66.7%) ^**a**^	18 (60%) ^**a**^	62 (62%) ^**a**^	0.48
Female	9 (37.5%)^**a**^	3 (16.7%) ^**a**^	6 (33.3%) ^**a**^	12 (40%) ^**a**^	38 (38%) ^**a**^	
**Age**Mean ±SD	43.7**± 1**4.2^**a**^	41.6**± 1**5.4^**a**^	40 **± 1**5^**a**^	51 **± 1**0^**b**^	46.7± 14.5^**a**^	0.005*
**Abdo. Pain No (%)**	19 (79.16%)^a^	17 (94.4%)^a^	10 (55.5%)^b^	21 (70%)^a^	75 (75%)^a^	0.016*
**Diarrhea No (%)**	4 (16.7%)	1 (5.6%)	3 (16.7%)	1 (3.3%)	2(2%)	0.13
**Constipation No (%)**	12 (50.0%)	17 (94.4%)	10 (55.5%)	15 (50.0%)	50 (50%)	0.21
**Alternating bowel habits No (%)**	12 (50.0%)	15 (83.3%)	14 (77.8%)	15 (50.0%)	20 (20%)	0.34
**Bleeding per rectum No (%)**	8 (33.3%)	13 (72.2%)	11 (61.1%)	13 (43.3%)	52 (52%)	0.267
**Mucous No (%)**	2 (8.3%)^a^	7 (38.9%)^b^	5 (27.8%)^b^	7 (23.3%)^b^	35 (35%)^b^	0.002*
**Weight loss No (%)**	5 (20.8%)^a^	9 (50.0%)^b^	1 (5.6%)^c^	9 (30.0%)^a^	44 (44%)^b^	0.05*
**Anemia No (%)**	11 (45.8%)^a^	18 (100%)^b^	11 (61.1%)^c^	18 (60.0%)^c^	70 (70%)^c^	0.015*

*p-value <0.05 is considered significant

Groups bearing different initials are significantly different

### Differential expression of the studied miRNAs in the investigated groups compared to the control group in the training set

**Fig 1:**
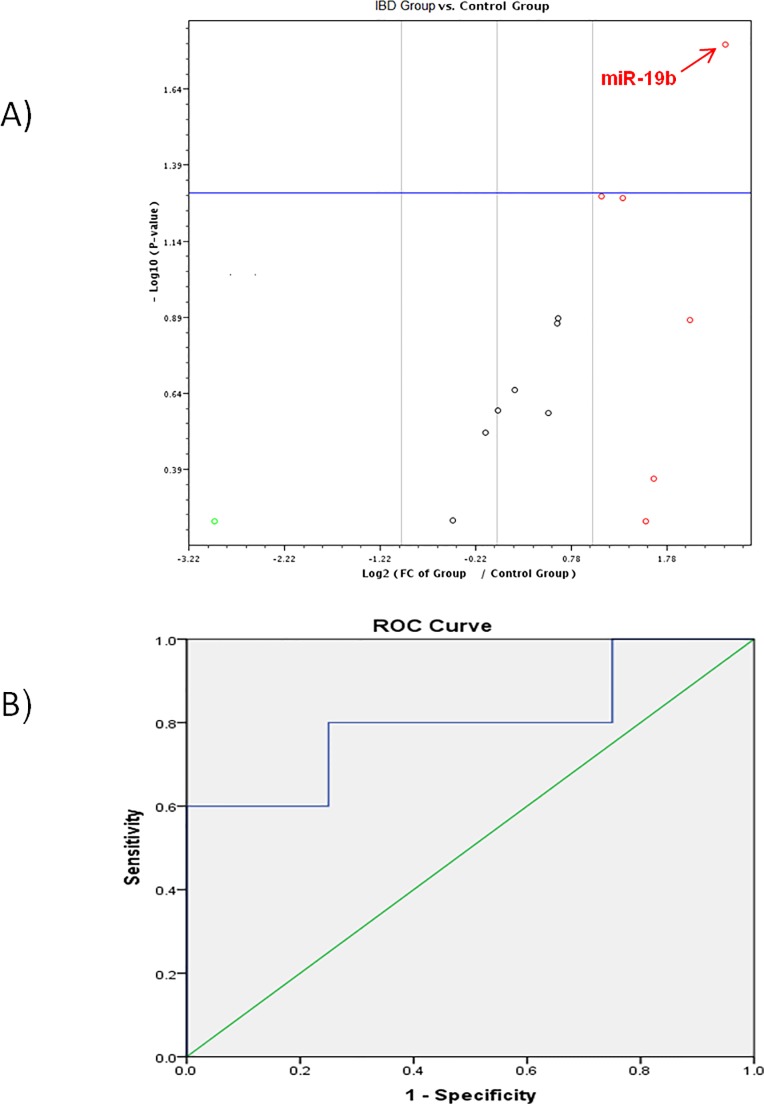
A) Volcano plot shows differential expression of 14-miRNAs in the IBD group versus the control group. *miR-19b* was significantly up regulated. B) ROC curve analysis of *miR-19b* between the IBD group and the control group.

**Fig 2:**
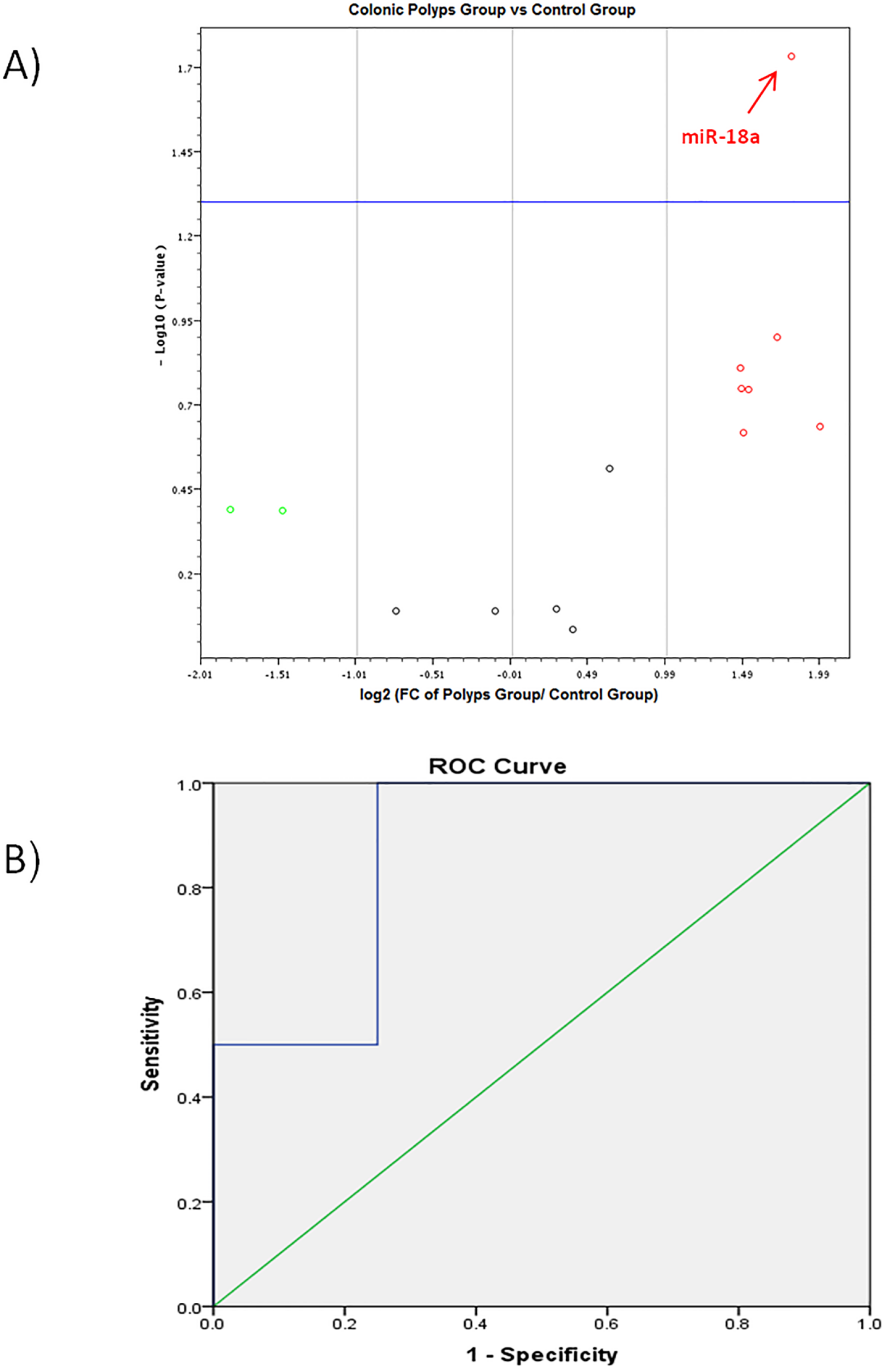
A) Volcano plot shows differential expression of 14-miRNAs in the colonic polyps group versus the control group. *miR-18a* was significantly up regulated. B) ROC curve analysis of *miR-18a* between the colonic polyps group and the control group.

**Fig 3:**
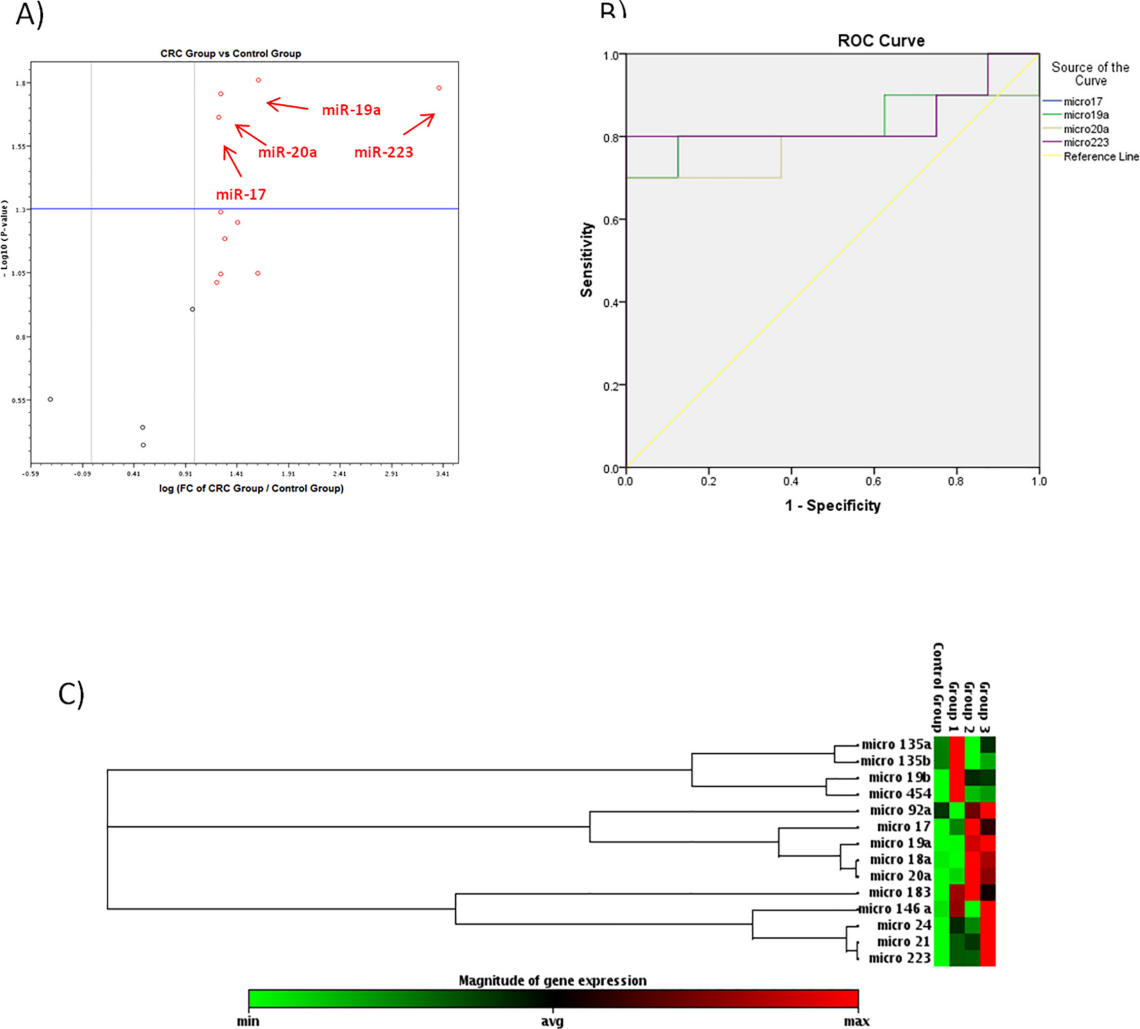
Volcano plot shows differential expression of 14-miRNAs in the CRC group versus the control group. *miR-17*,*miR-19a*.*miR-20a and miR-233* were significantly up regulated. B) ROC curve analysis of *miR-17*,*miR-19a*.*miR-20a and miR-233* between the CRC group and the control group. C) The clustergram performs hierarchical clustering of the entire dataset to display a heat map with dendrograms indicating co-regulated genes across control, IBD, colonic polyps and CRC groups respectively.

Among all studied miRNAs, only *miR-19b* was significantly up-regulated in the IBD group (fold change = 5.24, p-value = 0.016) **(Fig 1 and Table 2)**,whereas *miR-18a* was significantly up regulated in the CP group (fold change = 3.49, p-value = 0.018) **(Fig 2 and Table 3)** and *miR-17*, *miR-19a*, *miR-20a and miR-223* were significantly up-regulated in the CRC group (fold change = 2.35, 3.07, 2.38 & 10.35 and p-value = 0.021, 0.015, 0.017& 0.0166 respectively) as shown in **(Fig 3**and **Table 4).**

**Table 2:** Differential expression of the studied miRNAs in IBD group compared to the control group.

Gene Symbol	Fold change	p-value	95% CI
***miR-17***	1.5437	0.134724	(0.00001, 4.73)
***miR-18a***	0.9184	0.307535	(0.00001, 2.53)
***miR-19a***	1.0087	0.25978	(0.00001, 2.88)
***miR-19b***	5.2422	0.016254	(0.00001, 11.88)
***miR-20a***	1.1348	0.222622	(0.00001, 3.41)
***miR-21***	1.4549	0.26423	(0.00001, 3.70)
***miR-92a***	0.7227	0.597156	(0.00001, 2.11)
***miR-135a***	2.9328	0.601251	(0.00001, 9.64)
***miR-135b***	3.1227	0.434503	(0.00001, 11.73)
***miR-146 a***	2.1329	0.051392	(0.00001, 5.77)
***miR-183***	2.4807	0.051877	(0.00001, 7.14)
***miR-223***	4.0599	0.130626	(0.00001, 13.96)
***miR-454***	3.1887	0.213131	(0.00001, 9.89)
***miR-24***	1.5593	0.129352	(0.00001, 4.61)

**Table 3:** Differential expression of the studied miRNAs in colonic polyps compared to control group.

Gene Symbol	Fold change	p-value	95% CI
***miR-17***	3.278	0.126038	(0.00001, 7.29)
***miR-18a***	3.4941	0.018516	(0.99, 6.00)
***miR-19a***	2.8913	0.179212	(0.00001, 6.41)
***miR-19b***	2.7919	0.177971	(0.00001,6.14)
***miR-20a***	2.7814	0.155419	(0.00001, 6.29)
***miR-21***	1.5461	0.30692	(0.08, 3.01)
***miR-92a***	1.221	0.80316	(0.00001, 2.46)
***miR-135a***	0.3574	0.409282	(0.00001, 1.03)
***miR-135b***	0.2829	0.40799	(0.00001, 1.01)
***miR-146 a***	0.9267	0.816231	(0.12, 1.73)
***miR-183***	2.8224	0.241906	(0.00001, 7.29)
***miR-223***	3.9796	0.231098	(0.00001, 12.50)
***miR-454***	1.2779	0.860551	(0.00001, 3.47)
***miR-24***	1.3154	0.923229	(0.37, 2.26)

**Table 4:** Differential expression of the studied miRNAs in CRC group compared to control group.

Gene Symbol	Fold change	p-value	95% CI
***miR-17***	2.3519	0.021798	(0.19, 4.51)
***miR-18a***	3.0616	0.089514	(0.46, 5.66)
***miR-19a***	3.073	0.015544	(0.00001, 6.55)
***miR-19b***	2.6732	0.054453	(0.00001, 5.54)
***miR-20a***	2.385	0.017565	(0.00001, 4.85)
***miR-21***	2.3866	0.090037	(0.15, 4.62)
***miR-92a***	1.4162	0.426261	(0.00001, 3.14)
***miR-135a***	1.4117	0.36228	(0.00001, 3.97)
***miR-135b***	0.7578	0.281548	(0.00001, 2.61)
***miR-146 a***	2.4498	0.065661	(0.00001, 5.02)
***miR-183***	1.9738	0.124567	(0.00001, 4.78)
***miR-223***	10.359	0.016657	(0.00001, 33.69)
***miR-454***	1.4234	0.646062	(0.00001, 3.50)
***miR-24***	2.3268	0.097576	(0.00001, 4.74)

Upon comparing the CRC group (malignant) versus the other groups (non-malignant); only miR-223 was significantly up regulated (fold change = 4.49, p-value = 0.002) as shown in **(Table 5)**.

**Table 5:** Differential expression of the studied miRNAs in CRC group compared to all other groups.

Gene Symbol	Fold change	p-value	95%CI
***miR-17***	1.446	0.634455	(0.00001, 2.92)
***miR-18a***	2.158	0.087028	(0.24, 4.07)
***miR-19a***	2.2289	0.539639	(0.00001, 4.92)
***miR-19b***	1.1951	0.949393	(0.00001, 2.50)
***miR-20a***	1.6894	0.461582	(0.00001, 3.57)
***miR-21***	1.8714	0.365321	(0.28, 3.46)
***miR-92a***	1.4704	0.680683	(0.00001, 3.06)
***miR-135a***	1.392	0.952114	(0.00001, 3.27)
***miR-135b***	0.7865	0.545311	(0.00001, 2.06)
***miR-146 a***	1.997	0.482002	(0.00001, 4.09)
***miR-183***	1.1009	0.112391	(0.00001, 2.51)
***miR-223***	4.4959	0.00239	(0.00001, 10.82)
***miR-454***	0.9339	0.278063	(0.00001, 1.98)
***miR-24***	2.1874	0.186678	(0.00001, 3.79)

However, only *miR-135b* and *miR-454* were significantly down regulated in the CRC group compared to the IBD group (fold change = 0.24 & 0.44 and p-value = 0.023 & 0.018 respectively) as shown in **S2 Table**. While *miR-135a* and *miR-135b* were significantly down-regulated in the CP group compared to the IBD group (fold change = 0.12, 0.09 and p-value = 0.019 and 0.018 respectively) as shown in **S3 Table.**

### Differential expression of the studied miRNAs in the CRC group compared to the control group in the validation set

Among all studied miRNAs, only *miR-223* was significantly up-regulated in the CRC group (fold change = 4.06, p-value = 0.04) as shown in (**Table 6).** Upon splitting the CRC patients of the validation set into male and female groups; we found that *miR-19a*, *miR-19b*, and *miR-20a* were significantly up regulated in the female CRC patients compared to the control group (fold change = 2.8, 4.1 & 2.2 and p-value = 0.021, 0.023 & 0.04 respectively) as shown in **S4 Table**. On the other hand, only *miR-17* was significantly up-regulated in the male CRC patients compared to the control group (fold change = 2.1, p-value = 0.03) as shown in **S5 Table.**

**Table 6:** Differential expression of the studied miRNAs in CRC validation set versus control group.

Gene Symbol	Fold change	p-value	95%CI
***miR-17***	1.962	0.382051	(0.15, 3.77)
***miR-18a***	1.9561	0.145249	(0.71, 3.20)
***miR-19a***	2.6689	0.071306	(0.29, 5.04)
***miR-19b***	2.9433	0.174359	(0.26, 5.63)
***miR-20a***	1.9596	0.341793	(0.24, 3.68)
***miR-21***	1.4369	0.54292	(0.15, 2.72)
***miR-92a***	0.7756	0.551967	(0.01, 1.54)
***miR-135a***	1.5393	0.457262	(0.00001, 4.17)
***miR-135b***	0.6059	0.090761	(0.00001, 2.15)
***miR-146 a***	1.6258	0.088477	(0.36, 2.89)
***miR-183***	0.8923	0.617333	(0.00001, 2.00)
***miR-223***	4.069	0.042002	(0.00001, 22.91)
***miR-454***	1.5726	0.342075	(0.00001, 3.71)
***miR-24***	3.1791	0.389579	(0.30, 6.06)

### ROC curve analysis of differentially expressed miRNAs

For discriminating the CRC group from the control group; we have found that *miR-223* (AUC 0.838), miR-17 (AUC 0.813), *miR-19a* (AUC 0.825) possessed higher diagnostic performance than *miR-20a* (AUC 0.788). On the other hand, *miR-18a* (AUC 0.8) showed good diagnostic performance in discriminating the IBD group from the control group. Also, *miR-19b* (AUC 0.875) showed good diagnostic performance in the discriminating the CP group from the control group as shown in (**Table 7**).

**Table 7:** ROC curve analysis of individual miRNAs among the studied groups.

Compared groups	Test Result Variable(s)	AUC	Std. Errorr	95% Confidence Interval	
				Lower Bound	Upper Bound
**IBD vs Control**	***miR-19b***	800	144	517	1.000
**Colonic Polyps vs Control**	***miR-18a***	875	097	685	1.000
**CRC vs Control**	***miR-17***	813	114	589	1.000
	***miR-19a***	825	109	611	1.000
	***miR-20a***	788	117	558	1.000
	***miR-223***	838	108	627	1.000
**CRC vs IBD**	***miR-135b***	380	180	028	732
	***miR-454***	640	191	266	1.000

## Discussion

Our study assessed the possibility of using different circulating miRNAs as serological biomarkers for detection of CRC. Our study was properly designed to include cases at different stages of colorectal related diseases i.e. patients with inflammatory bowel disease (IBD), adenomatous colonic polyps (CP) and CRC.

Our results revealed that there was significant up regulation of *miR-17*, *miR-19a*, *miR-20a* and *miR-223* in the CRC group compared to the control group. The *miR-17*, *miR-20a and miR-19a* are members of the polycistronic *miR17-92* cluster (*miR-17*, *miR-18a*, *miR-19a*, *miR-19b*, *miR-20a*, *and miR-92a*) which located on 13q31.3; a locus that is frequently amplified in colorectal cancer [13, 14]. Our finding regarding significant up-regulation of *miR-20a* came in accordance with the previous results by Xu et al., who found that up-regulation of *miR-20* was associated with tumor invasion and lymph node metastasis of HCC patients [15]. So, *miR-20a* may possess a potential tumor oncogenic function in CRC. In addition, *miR-20a* promotes epithelial to mesenchymal transition (EMT), a key step in tumor metastasis, through targeting the tumor suppressor gene (tissue inhibitor matrix metalloproteinase 3 (TIMP3)); negative regulator of matrix metalloproteinase 2 (MMP2) and matrix metalloproteinase 9 (MMP9) that mediated extracellular matrix (ECM) degradation [15]. Also, miR-20a targets the activating members of the *E2F* family, including cell cycle and apoptosis regulatory genes, leading to G1- S phase transition. However, high levels of E2F proteins, especially E2F1, can induce apoptosis which acts as an emergency brake for over proliferating cells. The oncogenic function of *miR-20a* was proposed to hinder this emergency brake function of E2F factors and hence stimulate tumor growth [16]. Moreover, *miR-20a* controls cell cycle progression through targeting cyclin-dependent kinase inhibitors, CDKN1A/p21; important regulator of G2/S transition [17]. Furthermore, miR-20a targets tumor suppressor PTEN; which is a negative regulator of oncogenic prosurvival PI3K\AKT pathway [18, 19]. Our data regarding the upregulation of *miR-17a* and *miR-19a* in CRC patients are comparable to previous reports in literature [9, 16–19]. Similarly to *miR-20a*, *miR-17a* was shown to promote cell cycle progression through targeting CDKN1A/p21 and E2F [16, 17], whereas the oncogenic potential of *miR-19a* is achieved mainly through targeting the PTEN; thereby promoting the oncogenic pro-survival pathway [9, 18–20].

Our data regarding *miR-223* over expression in CRC group are concordant with those of Huang *et al* who assessed the expression levels of miRNAs in the plasma of 100 CRC patients and in 37 patients with colonic adenomas compared to 59 healthy individuals. Among their panel, 12 miRNAs were up regulated, including *miR-223* [21]. Similarly, Li *et al*. reported that the overexpression of *miR-223* in the neoplastic colonic tissues compared to the adjacent non-cancerous ones [22]. The oncogenic function of *miR-223* in CRC was achieved through targeting FOXO1 [23] and RASA1 [24]. FOXO1 is a member of FOX transcription factors-O subfamily. It regulates the transcription activity of various genes including p21, and thus inhibits cell-cycle progression [25, 26].On the other hand, RASA1, a RAS signaling terminator, is associated with tumor progression [27]. Moreover, Li *et al*. showed that *miR-223* could be considered a poor prognostic marker since it's over expression correlates significantly with high TNM stage, nodal and distant metastases as well as with reduced overall survival rates [22]. Our data also showed that significant up regulation of *miR-18a* in patients with CP compared to the control group. Our results supported a previous report by Wu et al., [28] who demonstrated that *miR-18a* plays an oncogenic role in colorectal carcinogenesis through down regulation of the Ataxia telangiectasia mutated gene (ATM); a key enzyme in the repair of DNA double-strand breaks. In addition, it phosphorylates several key proteins that initiate activation of the DNA damage checkpoint, leading to cell cycle arrest, DNA repair or apoptosis [29].

**Fig 4:**
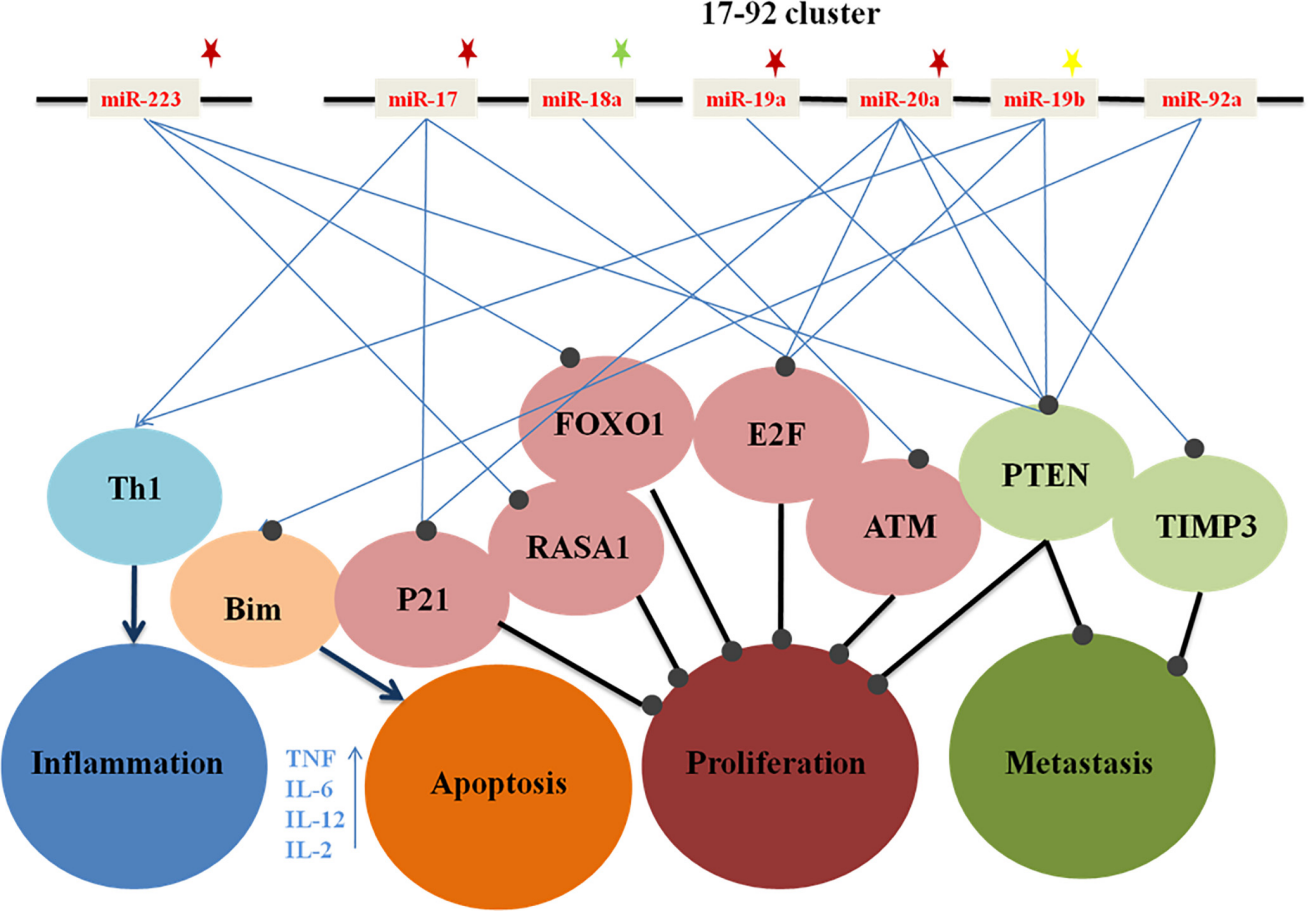
Network showing the interplay between significant miRNAs in CRC, IBD and colonic polyps group compared to control group. Red star indicates the significant miRNA in CRC while yellow star indicate significant miRNA in IBD and green star indicate miRNA in colonic polyps. The arrow indicates the activation while the circle at the end of line indicates inhibition.

As for *miR-19b*, significant up regulation was reported in patients with IBD compared to the control group. Jiang et al. showed that both *miR17* and *miR-19b*, belonging to *miR-17-92* cluster, inhibit Treg cell differentiation and promote Th1 responses [30]. Th1 is responsible for the pro inflammatory response via secreting of IL12, IL2, IL6, IFN and TNF [31]. The latter (TNF) plays a vital role in the inflammation process through activation of NF-kB pathway; a prototypical pro inflammatory signaling pathway [32]. The possible mechanistic role of significant miRNAs in the CRC, IBD and colonic polyps groups compared to the control group and their downstream targets were illustrated in the suggested cartoon **(Fig 4).**

Red star indicates the significant miRNA in CRC while yellow star indicates significant miRNA in IBD and green star indicates miRNA in colonic polyps. The arrow indicates the activation while the circle at the end of line indicates inhibition.

Further analysis of the data using (ROC) analysis curve and the corresponding area under the curve were attempted for the significant miRNAs in each pairwise comparison to investigate their diagnostic accuracy. ROC curve analysis of individual miRNAs revealed that *miR-17*, *miR-19a*, *miR-20a & miR-223* can effectively differentiate between the CRC group and the control group. However, *miR-18a* showed good diagnostic accuracy in discriminating the CP group from the control group. On the other hand, *miR-19b* showed good diagnostic accuracy in discriminating the IBD group from the control group.

### In conclusion

Aberrant miRNA expressions are highly involved in the cascade of colorectal carcinogenesis. We have found that (*miR-17*, *miR-19a*, *miR-20a* and *miR-223*) could be used as diagnostic biomarkers for CRC. On the other hand, *miR-19b* and *miR-18a* could be used as diagnostic biomarkers for CP and IBD respectively.

## Supporting Information

S1 Table The rational for choosing the studied 14 miRNAs based on prior references. (DOC)

S2 Table Differential expression of the studied miRNAs in CRC group versus IBD group. (DOC)

S3 Table Differential expression of the studied miRNAs in CP group versus IBD group. (DOC)

S4 Table Differential expression of the studied miRNAs in female CRC group versus control group "Validation Set". (DOC)

S5 Table Differential expression of the studied miRNAs in male CRC patients versus control group "Validation Set". (DOC)
