# Supplementary material for: Circulating Serum miRNAs as Diagnostic Markers for Colorectal Cancer
Source: PLoS One. 2016 May 2;11(5):e0154130. doi: 10.1371/journal.pone.0154130 (PMC4852935; doi:10.1371/journal.pone.0154130)
Supplement: S1 Table — (DOC) [file pone.0154130.s001.doc]

**S1 Table: The rational for choosing the studied 14 miRNAs based on prior references**

| **Gene Symbol** | **Putative mRNA targets** | **References** |
| --- | --- | --- |
| **micro 17**  (has been detected in plasma, feces and tissue ) **(Ref. 1-9**) | **P21, E2F** | 1. **Hofsli E**, Sjursen W, Prestvik WS, et al. Identification of serum microRNA profiles in colon cancer. Br J Cancer 2013; 108:1712-1719. 2. **Corté H**, Manceau G, Blons H, et al. MicroRNA and colorectal cancer. Dig Liver Dis .2012; 44:195–200. 3. [**Koga**](http://cancerprevres.aacrjournals.org/search?author1=Yoshikatsu+Koga&sortspec=date&submit=Submit) **Y**, [Yasunaga](http://cancerprevres.aacrjournals.org/search?author1=Masahiro+Yasunaga&sortspec=date&submit=Submit) M,  [Takahashi](http://cancerprevres.aacrjournals.org/search?author1=Amane+Takahashi&sortspec=date&submit=Submit) A,  [Kuroda](http://cancerprevres.aacrjournals.org/search?author1=Junichiro+Kuroda&sortspec=date&submit=Submit) J, et al. MicroRNA Expression Profiling of Exfoliated Colonocytes Isolated from Feces for Colorectal Cancer Screening. Cancer Prev Res .2010; 3: 1435. 4. **Ng EK**, Chong WW, Jin H, et al. Differential expression of microRNAs in plasma of patients with colorectal cancer: a potential marker for colorectal cancer screening. Gut 2009; 58:1375–81.   **Motoyama K**, Inoue H, Takatsuno Y, et al. Over- and under-expressed microRNAs in human colorectal cancer. Int J Oncol .2009; 34:1069–75.  **Nagel R**, le Sage C, Diosdado B, et al. Regulation of the adenomatous polypsosis coli gene by the miR-135 family in colorectal cancer. Cancer Res. 2008; 68:5795–802.   1. **Ivanovska I**, Ball AS, Diaz RL, et al. MicroRNAs in the miR-106b family regulate p21/CDKN1A and promote cell cycle progression. Mol Cell Biol .2008; 28: 2167–2174. 2. **Woods K**, Thomson JM, Hammond SM. Direct regulation of an oncogenic micro-RNA cluster by E2F transcription factors. J Biol Chem. 2007; 282: 2130–2134. 3. **Volinia S**, Calin GA, Liu CG, et al. A microRNA expression signature of human solid tumors defines cancer gene targets. Proc Natl Acad Sci USA .2006; 103:2257–61. |
| **micro 18a**  (has been detected in plasma, feces and tissue )  **(Ref.10-18)** | **ATM** | 1. **Ren A**, Dong Y, Tsoi H, et al. Detection of miRNA as non-invasive biomarkers of colorectal cancer. Int J Mol Sci, 2015;16:2810–2823. 2. **Wang J,** Song Y-X, Ma B, et al.Regulatory Roles of Non-Coding RNAs in Colorectal Cancer. Int J Mol Sci. 2015; 16:19886–919. 3. **Wu CW**, Dong YJ, Liang QY, et al. MicroRNA-18a attenuates DNA damage repair through suppressing the expression of ataxia telangiectasia mutated in colorectal cancer. PLoS ONE .2013; 8: e57036. 4. **Zhang G-J**, Zhou T, Liu Z-L, et al. Plasma miR-200c and miR-18a as potential biomarkers for the detection of colorectal carcinoma. Mol Clin Oncol .2013, 1:379–384. 5. **Corté H**, Manceau G, Blons H, et al. MicroRNA and colorectal cancer. Dig Liver Dis .2012, 44:195–200. 6. **Koga Y**, Yasunaga M., Takahashi A, et al. MicroRNA expression profiling of exfoliated colonocytes isolated from feces for colorectal cancer screening. Cancer Prev Res. 2010. 3: 1435–1442. 7. **Van Haaften G**, Agami R. Tumorigenicity of the miR-17-92 cluster distilled. Genes Dev, 2010; 24:1–4. 8. **Motoyama K**, Inoue H, Takatsuno Y, et al. Over- and under-expressed microRNAs in human colorectal cancer. Int J Oncol .2009; 34:1069–75. 9. **Banin S**, Moyal L, Shieh S, et al. Enhanced phosphorylation of p53 by ATM in response to DNA damage. Science. 1998; 281: 1674–7. |
| **micro 19a** (has been detected in serum, feces and tissue )  **(Ref.19-25)** | **PTEN** | 1. **Ren A**, Dong Y, Tsoi H, et al. Detection of miRNA as non-invasive biomarkers of colorectal cancer. Int J Mol Sci, 2015;16:2810–2823. 2. **Wang J,** Song Y-X, Ma B, et al.Regulatory Roles of Non-Coding RNAs in Colorectal Cancer. Int J Mol Sci. 2015; 16:19886–919. 3. **Zheng G**, Du L, Yang X, Zhang X, Wang L, Yang Y, et al. Serum microRNA panel as biomarkers for early diagnosis of colorectal adenocarcinoma. Br J Cancer 2014, 111:1985–92. 4. **Corté H**, Manceau G, Blons H, et al. MicroRNA and colorectal cancer. Dig Liver Dis .2012, 44:195–200. 5. **Koga Y**, Yasunaga M., Takahashi A, et al. MicroRNA expression profiling of exfoliated colonocytes isolated from feces for colorectal cancer screening. Cancer Prev Res. 2010, 3: 1435–1442. 6. **Van Haaften G**, Agami R. Tumorigenicity of the miR-17-92 cluster distilled. Genes Dev, 2010; 24:1–4.   **Arndt GM**, Dossey L, Cullen LM, Lai A, Druker R, Eisbacher M, et al. Characterization of global microRNA expression reveals oncogenic potential of miR-145 in metastatic colorectal cancer. BMC Cancer. 2009; 9:374. |
| **micro 19b** (has been detected in plasma and feces )  **(Ref.26-31)** | **PTEN, E2F** | 1. **Ren A**, Dong Y, Tsoi H, et al. Detection of miRNA as non-invasive biomarkers of colorectal cancer. Int J Mol Sci, 2015;16:2810–2823. 2. **Kalla R**, Ventham NT, Kennedy NA, et al. MicroRNAs: new players in IBD. Gut. 2014; 0:1–14. 3. **Giráldez MD**, Lozano JJ., Ramírez G, et al. Circulating microRNAs as biomarkers of colorectal cancer: Results from a genome-wide profiling and validation study. Clin Gastroenterol Hepatol. 2013, 11: 681–688. 4. **Corté H**, Manceau G, Blons H, et al. MicroRNA and colorectal cancer. Dig Liver Dis .2012, 44:195–200. 5. **Koga Y**, Yasunaga M., Takahashi A, et al. MicroRNA expression profiling of exfoliated colonocytes isolated from feces for colorectal cancer screening. Cancer Prev Res. 2010, 3: 1435–1442. 6. **Van Haaften G**, Agami R. Tumorigenicity of the miR-17-92 cluster distilled. Genes Dev, 2010; 24:1–4. |
| **micro 20a** (has been detected in plasma, tissue and feces )  **(Ref.32-42)** | **P21, E2F, PTEN, TIMP3** | 1. **Ren A**, Dong Y, Tsoi H, et al. Detection of miRNA as non-invasive biomarkers of colorectal cancer. Int J Mol Sci, 2015;16:2810–2823. 2. **Wang J,** Song Y-X, Ma B, et al.Regulatory Roles of Non-Coding RNAs in Colorectal Cancer. Int J Mol Sci. 2015; 16:19886–919. 3. **Xu T**, Jing C, Shi Y, et al. microRNA-20a enhances the epithelial-to-mesenchymal transition of colorectal cancer cells by modulating matrix metalloproteinases. Exp Ther Med. 2015; 10:683–688. 4. **Ahmed FE**, Ahmed NC, Vos PW, et al. Diagnostic microRNA markers to screen for sporadic human colon cancer in stool: I. Proof of Principle. Cancer Genomics Proteomics. 2013, 10: 93–113. 5. **Luo X**, Stock C, Burwinkel B, et al. Identification and evaluation of plasma microRNAs for early detection of colorectal cancer. PLoS ONE. 2013; 8: e62880. 6. **Koga Y**, Yasunaga M., Takahashi A, et al. MicroRNA expression profiling of exfoliated colonocytes isolated from feces for colorectal cancer screening. Cancer Prev Res. 2010, 3: 1435–1442. 7. **Corté H**, Manceau G, Blons H, et al. MicroRNA and colorectal cancer. Dig Liver Dis. 2012; 44:195–200. 8. **Van Haaften G**, Agami R. Tumorigenicity of the miR-17-92 cluster distilled. Genes Dev, 2010; 24:1–4. 9. **Motoyama K**, Inoue H, Takatsuno Y, et al. Over- and under-expressed microRNAs in human colorectal cancer. Int J Oncol. 2009; 34:1069–75. 10. **Woods K**, Thomson JM, Hammond SM. Direct regulation of an oncogenic micro-RNA cluster by E2F transcription factors. J Biol Chem. 2007; 282: 2130–2134. 11. **Volinia S**, Calin GA, Liu CG, et al. A microRNA expression signature of human solid tumors defines cancer gene targets. Proc Natl Acad Sci USA. 2006; 103:2257–61. |
| **micro 21** (has been detected in plasma and serum )  **(Ref.43-52)** | **PDCD4, RASA1** | 1. [**Yu W**](http://www.ncbi.nlm.nih.gov/pubmed/?term=Yu W%5BAuthor%5D&cauthor=true&cauthor_uid=26893868), [Wang Z](http://www.ncbi.nlm.nih.gov/pubmed/?term=Wang Z%5BAuthor%5D&cauthor=true&cauthor_uid=26893868), [Shen LI](http://www.ncbi.nlm.nih.gov/pubmed/?term=Shen LI%5BAuthor%5D&cauthor=true&cauthor_uid=26893868), et al. Circulating microRNA-21 as a potential diagnostic marker for colorectal cancer: A meta-analysis. [Mol Clin Oncol.](http://www.ncbi.nlm.nih.gov/pubmed/26893868) 2016; 4(2):237-244. 2. [**Gong B**](http://www.ncbi.nlm.nih.gov/pubmed/?term=Gong B%5BAuthor%5D&cauthor=true&cauthor_uid=25663768), [Liu WW](http://www.ncbi.nlm.nih.gov/pubmed/?term=Liu WW%5BAuthor%5D&cauthor=true&cauthor_uid=25663768), [Nie WJ](http://www.ncbi.nlm.nih.gov/pubmed/?term=Nie WJ%5BAuthor%5D&cauthor=true&cauthor_uid=25663768), et al. MiR-21/RASA1 axis affects malignancy of colon cancer cells via RAS pathways. [World J Gastroenterol.](http://www.ncbi.nlm.nih.gov/pubmed/25663768) 2015; 21(5):1488-97. 3. **Ren A**, Dong Y, Tsoi H, et al. Detection of miRNA as non-invasive biomarkers of colorectal cancer. Int J Mol Sci, 2015;16:2810–2823. 4. [**Xu F**](http://www.ncbi.nlm.nih.gov/pubmed/?term=Xu F%5BAuthor%5D&cauthor=true&cauthor_uid=25683351), [Xu L](http://www.ncbi.nlm.nih.gov/pubmed/?term=Xu L%5BAuthor%5D&cauthor=true&cauthor_uid=25683351), [Wang M](http://www.ncbi.nlm.nih.gov/pubmed/?term=Wang M%5BAuthor%5D&cauthor=true&cauthor_uid=25683351), et al. The accuracy of circulating microRNA-21 in the diagnosis of colorectal cancer: a systematic review and meta-analysis. [Colorectal Dis.](http://www.ncbi.nlm.nih.gov/pubmed/25683351) 2015; 17(5):O100-7. 5. **Wang J,** Song Y-X, Ma B, et al.Regulatory Roles of Non-Coding RNAs in Colorectal Cancer. Int J Mol Sci. 2015; 16:19886–919. 6. [**Basati G**](http://www.ncbi.nlm.nih.gov/pubmed/?term=Basati G%5BAuthor%5D&cauthor=true&cauthor_uid=25178939), [Emami Razavi A](http://www.ncbi.nlm.nih.gov/pubmed/?term=Emami Razavi A%5BAuthor%5D&cauthor=true&cauthor_uid=25178939), [Abdi S](http://www.ncbi.nlm.nih.gov/pubmed/?term=Abdi S%5BAuthor%5D&cauthor=true&cauthor_uid=25178939), [et](http://www.ncbi.nlm.nih.gov/pubmed/?term=Mirzaei A%5BAuthor%5D&cauthor=true&cauthor_uid=25178939) al. Elevated level of microRNA-21 in the serum of patients with colorectal cancer. [Med Oncol.](http://www.ncbi.nlm.nih.gov/pubmed/25178939) 2014; 31(10):205. 7. **Ahmed FE**, Amed NC, Vos PW, et al. Diagnostic microRNA markers to screen for sporadic human colon cancer in blood. Cancer Genomics Proteomics. 2012; 9: 179–192. 8. **Corté H**, Manceau G, Blons H, et al. MicroRNA and colorectal cancer. Dig Liver Dis. 2012; 44:195–200. 9. **Schee K**, Boye K, Abrahamsen TW, et al. Clinical relevance of microRNA miR-21, miR-31, miR-92a, miR-101, miR-106a and miR-145 in colorectal cancer. BMC Cancer. 2012, 12: 505. 10. **Asangani IA**, Rasheed SA, Nikolova DA, et al. MicroRNA-21 (miR-21) post-transcriptionally downregulates tumor suppressor Pdcd4 and stimulates invasion, intravasation and metastasis in colorectal cancer. Oncogene. 2008; 27: 2128–2136. |
| **micro 92a** (has been detected in plasma, serum and tissue )  **(Ref.53-66)** | **PTEN, BIM** | 1. **Ren A**, Dong Y, Tsoi H, et al. Detection of miRNA as non-invasive biomarkers of colorectal cancer. Int J Mol Sci, 2015;16:2810–2823. 2. **Wang J,** Song Y-X, Ma B, et al.Regulatory Roles of Non-Coding RNAs in Colorectal Cancer. Int J Mol Sci. 2015; 16:19886–919. 3. **Wang J**, Huang S, Zhao M, et al. Identification of a circulating microRNA signature for colorectal cancer detection. PLoS One**.** 2014, 9: e87451. 4. **Yang X**, Zeng Z, Hou Y, et al. MicroRNA-92a as a potential biomarker in diagnosis of colorectal cancer: A systematic review and meta-analysis. PLoS ONE .2014, 9: e88745. 5. **Zhang G**, Zhou H, Xiao H, et al. MicroRNA-92a functions as an oncogene in colorectal cancer by targeting PTEN. Dig Dis Sci. 2014; 59: 98–107. 6. **Zhou T**, Zhang G, Liu Z, et al. Overexpression of miR-92a correlates with tumor metastasis and poor prognosis in patients with colorectal cancer. Int J Colorectal Dis. 2013; 28: 19–24. 7. **Corté H**, Manceau G, Blons H, et al. MicroRNA and colorectal cancer. Dig Liver Dis. 2012; 44:195–200. 8. **Schee K**, Boye K, Abrahamsen TW, et al. Clinical relevance of microRNA miR-21, miR-31, miR-92a, miR-101, miR-106a and miR-145 in colorectal cancer. BMC Cancer. 2012, 12: 505. 9. **Wu CW**, Ng SSM, Dong YJ, et al. Detection of miR-92a and miR-21 in stool samples as potential screening biomarkers for colorectal cancer and polyps. Gut .2012; 61: 739–745. 10. **Slattery ML**, Wolff E, Hoffman MD, et al. MicroRNAs and colon and rectal cancer: differential expression by tumor location and subtype. Genes Chromosomes Cancer .2011; 50:196–206. 11. **Huang Z**, Huang D, Ni S, et al. Plasma microRNAs are promising novel biomarkers for early detection of colorectal cancer. Int J Cancer. 2010; 126:118–126. 12. **Motoyama K**, Inoue H, Takatsuno Y, et al. Over- and under-expressed microRNAs in human colorectal cancer. Int J Oncol .2009; 34:1069–75. 13. **Ng EK**, Chong WW, Jin H, et al. Differential expression of microRNAs in plasma of patients with colorectal cancer: a potential marker for colorectal cancer screening. Gut .2009; 58:1375–81. 14. **Schetter AJ**, Leung SY, Sohn JJ, et al. MicroRNA expression profiles associated with prognosis and therapeutic outcome in colon adenocarcinoma. J Am Med Assoc. 2008; 299:425–36. |
| **micro 135a** (has been detected in plasma, serum and tissue  **(Ref.67-72)** | **APC, MTSS1** | 1. **Ren A**, Dong Y, Tsoi H, et al. Detection of miRNA as non-invasive biomarkers of colorectal cancer. Int J Mol Sci, 2015;16:2810–2823. 2. **Corté H**, Manceau G, Blons H, et al. MicroRNA and colorectal cancer. Dig Liver Dis. 2012; 44:195–200. 3. [**Zhou W**](http://www.ncbi.nlm.nih.gov/pubmed/?term=Zhou W%5BAuthor%5D&cauthor=true&cauthor_uid=23017832), [Li X](http://www.ncbi.nlm.nih.gov/pubmed/?term=Li X%5BAuthor%5D&cauthor=true&cauthor_uid=23017832), [Liu F](http://www.ncbi.nlm.nih.gov/pubmed/?term=Liu F%5BAuthor%5D&cauthor=true&cauthor_uid=23017832), et al. MiR-135a promotes growth and invasion of colorectal cancer via metastasis suppressor 1 in vitro. [Acta Biochim Biophys Sin (Shanghai).](http://www.ncbi.nlm.nih.gov/pubmed/23017832) 2012; 44(10):838-46. 4. **Earle JS**, Luthra R, Romans A, et al. Association of microRNA expression with microsatellite instability status in colorectal adenocarcinoma. J Mol Diagn.2010; 12:433–40. 5. [**Nagel R**](http://www.ncbi.nlm.nih.gov/pubmed/?term=Nagel R%5BAuthor%5D&cauthor=true&cauthor_uid=18632633), [le Sage C](http://www.ncbi.nlm.nih.gov/pubmed/?term=le Sage C%5BAuthor%5D&cauthor=true&cauthor_uid=18632633), [Diosdado B](http://www.ncbi.nlm.nih.gov/pubmed/?term=Diosdado B%5BAuthor%5D&cauthor=true&cauthor_uid=18632633), et al. Regulation of the adenomatous polyposis coli gene by the miR-135 family in colorectal cancer. [Cancer Res.](http://www.ncbi.nlm.nih.gov/pubmed/18632633) 2008; 68(14):5795-802. 6. **Schetter AJ**, Leung SY, Sohn JJ, et al. MicroRNA expression profiles associated with prognosis and therapeutic outcome in colon adenocarcinoma. J Am Med Assoc. 2008; 299:425–36. |
| **micro 135b** (has been detected in plasma, serum and tissue  **(Ref.73-84)** | **TGFBR2, MTSS1, MTUS1** | 1. **He Y**, Wang J, Wang J, et al. MicroRNA-135b regulates apoptosis and chemoresistance in colorectal cancer by targeting large tumor suppressor kinase 2. [Am J Cancer Res.](http://www.ncbi.nlm.nih.gov/pubmed/26101704) 2015; 5(4):1382-95. 2. [**Li J**](http://www.ncbi.nlm.nih.gov/pubmed/?term=Li J%5BAuthor%5D&cauthor=true&cauthor_uid=26677848), [Liang H](http://www.ncbi.nlm.nih.gov/pubmed/?term=Liang H%5BAuthor%5D&cauthor=true&cauthor_uid=26677848), [Bai M](http://www.ncbi.nlm.nih.gov/pubmed/?term=Bai M%5BAuthor%5D&cauthor=true&cauthor_uid=26677848), et al. miR-135b Promotes Cancer Progression by Targeting Transforming Growth Factor Beta Receptor II (TGFBR2) in Colorectal Cancer. [PLoS One.](http://www.ncbi.nlm.nih.gov/pubmed/26677848) 2015; 10(12):e0145589. 3. [**Ozcan O**](http://www.ncbi.nlm.nih.gov/pubmed/?term=Ozcan O%5BAuthor%5D&cauthor=true&cauthor_uid=26643896), [Kara M](http://www.ncbi.nlm.nih.gov/pubmed/?term=Kara M%5BAuthor%5D&cauthor=true&cauthor_uid=26643896), [Yumrutas O](http://www.ncbi.nlm.nih.gov/pubmed/?term=Yumrutas O%5BAuthor%5D&cauthor=true&cauthor_uid=26643896), et al. MTUS1 and its targeting miRNAs in colorectal carcinoma: significant associations. [Tumour Biol.](http://www.ncbi.nlm.nih.gov/pubmed/26643896) 2015. [Epub ahead of print]. 4. **Ren A**, Dong Y, Tsoi H, et al. Detection of miRNA as non-invasive biomarkers of colorectal cancer. Int J Mol Sci, 2015;16:2810–2823. 5. **Wang J,** Song Y-X, Ma B, et al. Regulatory Roles of Non-Coding RNAs in Colorectal Cancer. Int J Mol Sci. 2015; 16:19886–919. 6. **Valeri N**, Braconi C, Gasparini P, et al. MicroRNA-135b promotes cancer progression by acting as a downstream effector of oncogenic pathways in colon cancer. Cancer Cell. 2014; 25:469–483. 7. **Wu CW**, Ng SC, Dong Y, et al. Identification of microRNA-135b in stool as a potential noninvasive biomarker for colorectal cancer and adenoma. Clin. Cancer Res. 2014; 20:2994–3002. 8. [**Wu W**](http://www.ncbi.nlm.nih.gov/pubmed/?term=Wu W%5BAuthor%5D&cauthor=true&cauthor_uid=24343340), [Wang Z](http://www.ncbi.nlm.nih.gov/pubmed/?term=Wang Z%5BAuthor%5D&cauthor=true&cauthor_uid=24343340), [Yang P](http://www.ncbi.nlm.nih.gov/pubmed/?term=Yang P%5BAuthor%5D&cauthor=true&cauthor_uid=24343340), et al. MicroRNA-135b regulates metastasis suppressor 1 expression and promotes migration and invasion in colorectal cancer. [Mol Cell Biochem.](http://www.ncbi.nlm.nih.gov/pubmed/24343340) 2014; 388:249-59. 9. **Khatri R**, Subramanian S. MicroRNA-135b and its circuitry networks as potential therapeutic targets in colon cancer. Front Oncol. 2013; 3:268. 10. **Corté H**, Manceau G, Blons H, et al. MicroRNA and colorectal cancer. Dig Liver Dis. 2012; 44:195–200. 11. [**Xu XM**](http://www.ncbi.nlm.nih.gov/pubmed/?term=Xu XM%5BAuthor%5D&cauthor=true&cauthor_uid=22844381), [Qian JC](http://www.ncbi.nlm.nih.gov/pubmed/?term=Qian JC%5BAuthor%5D&cauthor=true&cauthor_uid=22844381), [Deng ZL](http://www.ncbi.nlm.nih.gov/pubmed/?term=Deng ZL%5BAuthor%5D&cauthor=true&cauthor_uid=22844381), et al. Expression of miR-21, miR-31, miR-96 and miR-135b is correlated with the clinical parameters of colorectal cancer. [Oncol Lett.](http://www.ncbi.nlm.nih.gov/pubmed/22844381) 2012; 4(2):339-345. 12. **Ng EK**, Chong WW, Jin H, et al. Differential expression of microRNAs in plasma of patients with colorectal cancer: a potential marker for colorectal cancer screening. Gut 2009; 58:1375–81. |
| **micro 146 a** (has been detected in feces and tissue  **(Ref.85-88)** | Its target has not been identified yet | 1. [**Mao Y**](http://www.ncbi.nlm.nih.gov/pubmed/?term=Mao Y%5BAuthor%5D&cauthor=true&cauthor_uid=24740563), [Li Y](http://www.ncbi.nlm.nih.gov/pubmed/?term=Li Y%5BAuthor%5D&cauthor=true&cauthor_uid=24740563), [Jing F](http://www.ncbi.nlm.nih.gov/pubmed/?term=Jing F%5BAuthor%5D&cauthor=true&cauthor_uid=24740563), et al. Association of a genetic variant in microRNA-146a with risk of colorectal cancer: a population-based case-control study. [Tumour Biol.](http://www.ncbi.nlm.nih.gov/pubmed/24740563) 2014; 35(7):6961-7. 2. [**Zeng C**](http://www.ncbi.nlm.nih.gov/pubmed/?term=Zeng C%5BAuthor%5D&cauthor=true&cauthor_uid=24670457), [Huang L](http://www.ncbi.nlm.nih.gov/pubmed/?term=Huang L%5BAuthor%5D&cauthor=true&cauthor_uid=24670457), [Zheng Y](http://www.ncbi.nlm.nih.gov/pubmed/?term=Zheng Y%5BAuthor%5D&cauthor=true&cauthor_uid=24670457), et al. Expression of miR-146a in colon cancer and its significance. [Nan Fang Yi Ke Da Xue Xue Bao.](http://www.ncbi.nlm.nih.gov/pubmed/24670457) 2014; 34(3):396-400. 3. [**Ma XP**](http://www.ncbi.nlm.nih.gov/pubmed/?term=Ma XP%5BAuthor%5D&cauthor=true&cauthor_uid=24278149), [Zhang T](http://www.ncbi.nlm.nih.gov/pubmed/?term=Zhang T%5BAuthor%5D&cauthor=true&cauthor_uid=24278149), [Peng B](http://www.ncbi.nlm.nih.gov/pubmed/?term=Peng B%5BAuthor%5D&cauthor=true&cauthor_uid=24278149), et al. Association between microRNA polymorphisms and cancer risk based on the findings of 66 case-control studies. [PLoS One.](http://www.ncbi.nlm.nih.gov/pubmed/24278149) 2013; 8(11):e79584. 4. [**Ahmed FE**](http://www.ncbi.nlm.nih.gov/pubmed/?term=Ahmed FE%5BAuthor%5D&cauthor=true&cauthor_uid=23741026), [Ahmed NC](http://www.ncbi.nlm.nih.gov/pubmed/?term=Ahmed NC%5BAuthor%5D&cauthor=true&cauthor_uid=23741026), [Vos PW](http://www.ncbi.nlm.nih.gov/pubmed/?term=Vos PW%5BAuthor%5D&cauthor=true&cauthor_uid=23741026), et al. Diagnostic microRNA markers to screen for sporadic human colon cancer in stool: I. Proof of principle. [Cancer Genomics Proteomics.](http://www.ncbi.nlm.nih.gov/pubmed/23741026) 2013; 10(3):93-113. |
| **micro 183** (has been detected in plasma, serum, feces and tissue)  **(Ref.89-97)** | **MTUS1, Sox2, Klf4, UVRAG** | 1. [**Huangfu L**](http://www.ncbi.nlm.nih.gov/pubmed/?term=Huangfu L%5BAuthor%5D&cauthor=true&cauthor_uid=26717041), [Liang H](http://www.ncbi.nlm.nih.gov/pubmed/?term=Liang H%5BAuthor%5D&cauthor=true&cauthor_uid=26717041), [Wang G](http://www.ncbi.nlm.nih.gov/pubmed/?term=Wang G%5BAuthor%5D&cauthor=true&cauthor_uid=26717041), et al. miR-183 regulates autophagy and apoptosis in colorectal cancer through targeting of UVRAG. [Oncotarget.](http://www.ncbi.nlm.nih.gov/pubmed/26717041) 2015. [Epub ahead of print]. 2. [**Ozcan O**](http://www.ncbi.nlm.nih.gov/pubmed/?term=Ozcan O%5BAuthor%5D&cauthor=true&cauthor_uid=26643896), [Kara M](http://www.ncbi.nlm.nih.gov/pubmed/?term=Kara M%5BAuthor%5D&cauthor=true&cauthor_uid=26643896), [Yumrutas O](http://www.ncbi.nlm.nih.gov/pubmed/?term=Yumrutas O%5BAuthor%5D&cauthor=true&cauthor_uid=26643896), et al. MTUS1 and its targeting miRNAs in colorectal carcinoma: significant associations. [Tumour Biol.](http://www.ncbi.nlm.nih.gov/pubmed/26643896) 2015. [Epub ahead of print]. 3. **Ren A**, Dong Y, Tsoi H, et al. Detection of miRNA as non-invasive biomarkers of colorectal cancer. Int J Mol Sci, 2015;16:2810–2823. 4. **Wang J,** Song Y-X, Ma B, et al.Regulatory Roles of Non-Coding RNAs in Colorectal Cancer. Int J Mol Sci. 2015; 16:19886–919. 5. [**Yuan D**](http://www.ncbi.nlm.nih.gov/pubmed/?term=Yuan D%5BAuthor%5D&cauthor=true&cauthor_uid=25629978), [Li K](http://www.ncbi.nlm.nih.gov/pubmed/?term=Li K%5BAuthor%5D&cauthor=true&cauthor_uid=25629978), [Zhu K](http://www.ncbi.nlm.nih.gov/pubmed/?term=Zhu K%5BAuthor%5D&cauthor=true&cauthor_uid=25629978), et al. Plasma miR-183 predicts recurrence and prognosis in patients with colorectal cancer. [Cancer Biol Ther.](http://www.ncbi.nlm.nih.gov/pubmed/25629978) 2015; 16(2):268-75. 6. **Ahmed FE**, Ahmed NC, Vos PW, et al. Diagnostic microRNA markers to screen for sporadic human colon cancer in stool: I. Proof of Principle. Cancer Genomics Proteomics. 2013, 10: 93–113. 7. **Earle JS**, Luthra R, Romans A, et al. Association of microRNA expression with microsatellite instability status in colorectal adenocarcinoma. J Mol Diagn.2010; 12:433–40. 8. [**Wellner U**](http://www.ncbi.nlm.nih.gov/pubmed/?term=Wellner U%5BAuthor%5D&cauthor=true&cauthor_uid=19935649), [Schubert J](http://www.ncbi.nlm.nih.gov/pubmed/?term=Schubert J%5BAuthor%5D&cauthor=true&cauthor_uid=19935649), [Burk UC](http://www.ncbi.nlm.nih.gov/pubmed/?term=Burk UC%5BAuthor%5D&cauthor=true&cauthor_uid=19935649), et al. The EMT-activator ZEB1 promotes tumorigenicity by repressing stemness-inhibiting microRNAs. [Nat Cell Biol.](http://www.ncbi.nlm.nih.gov/pubmed/19935649) 2009; 11(12):1487-95. 9. **Motoyama K**, Inoue H, Takatsuno Y, et al. Over- and under-expressed microRNAs in human colorectal cancer. Int J Oncol .2009; 34:1069–75. |
| **micro 223** (has been detected in plasma, serum, feces and tissue)  **(Ref.98-108)** | **RASA1, FOXO1, PTEN** | 1. **Sun D**, Wang C, Long S, et al. C/EBP-β-activated microRNA-223 promotes tumour growth through targeting RASA1 in human colorectal cancer. Br J Cancer .2015; 112:1491–1500. 2. [**Li ZW**](http://www.ncbi.nlm.nih.gov/pubmed/?term=Li ZW%5BAuthor%5D&cauthor=true&cauthor_uid=25270282), [Yang YM](http://www.ncbi.nlm.nih.gov/pubmed/?term=Yang YM%5BAuthor%5D&cauthor=true&cauthor_uid=25270282), [Du LT](http://www.ncbi.nlm.nih.gov/pubmed/?term=Du LT%5BAuthor%5D&cauthor=true&cauthor_uid=25270282), et al. Overexpression of miR-223 correlates with tumor metastasis and poor prognosis in patients with colorectal cancer. Med Oncol. 2014; 31 (11): 256. 3. **Phua LC**, Chue XP, Koh, PK, et al. Global fecal microRNA profiling in the identification of biomarkers for colorectal cancer screening among Asians. Oncol Rep. 2014; 32: 97–104. 4. **Zhang J**, Luo X, Li H, et al. MicroRNA-223 functions as an oncogene in human colorectal cancer cells. Oncol Rep. 2014; 32:115–120. 5. **Zheng G**, Du L, Yang X, et al: Serum microRNA panel as biomarkers for early diagnosis of colorectal adenocarcinoma. Br J Cancer. 2014; 111:1985–92. 6. **Corté H**, Manceau G, Blons H, et al. MicroRNA and colorectal cancer. Dig Liver Dis. 2012; 44:195–200. 7. **Wu L**, Li H, Jia CY, et al. MicroRNA-223 regulates FOXO1 expression and cell proliferation. FEBS Lett. 2012; 586:1038–1043. 8. **Slattery ML**, Wolff E, Hoffman MD, et al. MicroRNAs and colon and rectal cancer: differential expression by tumor location and subtype. Genes Chromosomes Cancer 2011; 50:196–206. 9. **Huang Z**, Huang D, Ni S, et al. Plasma microRNAs are promising novel biomarkers for early detection of colorectal cancer. Int J Cancer. 2010; 127: 118-126. 10. **Schetter AJ**, Leung SY, Sohn JJ, et al. MicroRNA expression profiles associated with prognosis and therapeutic outcome in colon adenocarcinoma. J Am Med Assoc .2008; 299:425–36. 11. **Volinia S**, Calin GA, Liu CG, et al. A microRNA expression signature of human solid tumors defines cancer gene targets. Proc Natl Acad Sci USA .2006; 103:2257–61. |
| **micro 454** (has been detected in tissue)  **(Ref.109)** | **CYLD** | 1. [**Liang HL**](http://www.ncbi.nlm.nih.gov/pubmed/?term=Liang HL%5BAuthor%5D&cauthor=true&cauthor_uid=25824771), [Hu AP](http://www.ncbi.nlm.nih.gov/pubmed/?term=Hu AP%5BAuthor%5D&cauthor=true&cauthor_uid=25824771), [Li SL](http://www.ncbi.nlm.nih.gov/pubmed/?term=Li SL%5BAuthor%5D&cauthor=true&cauthor_uid=25824771), et al. MiR-454 prompts cell proliferation of human colorectal cancer cells by repressing CYLD expression. [Asian Pac J Cancer Prev.](http://www.ncbi.nlm.nih.gov/pubmed/25824771) 2015; 16(6):2397-402. |
| **micro 24** (has been detected in tissue and plasma)  **(Ref.110-113)** | **S phase enzyme** | 1. [**Fang Z**](http://www.ncbi.nlm.nih.gov/pubmed/?term=Fang Z%5BAuthor%5D&cauthor=true&cauthor_uid=26297223), [Tang J](http://www.ncbi.nlm.nih.gov/pubmed/?term=Tang J%5BAuthor%5D&cauthor=true&cauthor_uid=26297223), [Bai Y](http://www.ncbi.nlm.nih.gov/pubmed/?term=Bai Y%5BAuthor%5D&cauthor=true&cauthor_uid=26297223), et al. Plasma levels of microRNA-24, microRNA-320a, and microRNA-423-5p are potential biomarkers for colorectal carcinoma. [J Exp Clin Cancer Res.](http://www.ncbi.nlm.nih.gov/pubmed/26297223) 2015; 34:86. 2. [**Gattolliat CH**](http://www.ncbi.nlm.nih.gov/pubmed/?term=Gattolliat CH%5BAuthor%5D&cauthor=true&cauthor_uid=25586944), [Uguen A](http://www.ncbi.nlm.nih.gov/pubmed/?term=Uguen A%5BAuthor%5D&cauthor=true&cauthor_uid=25586944), [Pesson M](http://www.ncbi.nlm.nih.gov/pubmed/?term=Pesson M%5BAuthor%5D&cauthor=true&cauthor_uid=25586944), et al. MicroRNA and targeted mRNA expression profiling analysis in human colorectal adenomas and adenocarcinomas. [Eur J Cancer.](http://www.ncbi.nlm.nih.gov/pubmed/25586944) 2015; 51(3):409-20. 3. [**Gao Y**](http://www.ncbi.nlm.nih.gov/pubmed/?term=Gao Y%5BAuthor%5D&cauthor=true&cauthor_uid=25502080), [Liu Y](http://www.ncbi.nlm.nih.gov/pubmed/?term=Liu Y%5BAuthor%5D&cauthor=true&cauthor_uid=25502080), [Du L](http://www.ncbi.nlm.nih.gov/pubmed/?term=Du L%5BAuthor%5D&cauthor=true&cauthor_uid=25502080), et al. Down-regulation of miR-24-3p in colorectal cancer is associated with malignant behavior. [Med Oncol.](http://www.ncbi.nlm.nih.gov/pubmed/25502080) 2015; 32(1):362. 4. [**Mishra PJ**](http://www.ncbi.nlm.nih.gov/pubmed/?term=Mishra PJ%5BAuthor%5D&cauthor=true&cauthor_uid=20041160), [Song B](http://www.ncbi.nlm.nih.gov/pubmed/?term=Song B%5BAuthor%5D&cauthor=true&cauthor_uid=20041160), [Mishra PJ](http://www.ncbi.nlm.nih.gov/pubmed/?term=Mishra PJ%5BAuthor%5D&cauthor=true&cauthor_uid=20041160), et al. MiR-24 tumor suppressor activity is regulated independent of p53 and through a target site polymorphism. [PLoS One.](http://www.ncbi.nlm.nih.gov/pubmed/20041160) 2009; 4(12):e8445. |
