# Supplementary material for: Circulating Serum miRNAs as Diagnostic Markers for Colorectal Cancer
Source: PLoS One. 2016 May 2;11(5):e0154130. doi: 10.1371/journal.pone.0154130 (PMC4852935; doi:10.1371/journal.pone.0154130)
Supplement: S2 Table — (DOC) [file pone.0154130.s002.doc]

**S2 Table: Differential expression of the studied miRNAs in CRC group versus IBD group:**

| **Gene Symbol** | **Fold change** | **p-value** | **95% CI** |
| --- | --- | --- | --- |
| ***miR-17*** | 1.5235 | 0.326362 | (0.00001, 4.76) |
| ***miR-18a*** | 3.3337 | 0.286659 | (0.00001, 9.33) |
| ***miR-19a*** | 3.0463 | 0.810378 | (0.00001, 9.29) |
| ***miR-19b*** | 0.5099 | 0.27877 | (0.00001, 1.26) |
| ***miR-20a*** | 2.1018 | 0.687847 | (0.00001, 6.55) |
| ***miR-21*** | 1.6404 | 0.770995 | (0.00001, 4.19) |
| ***miR-92a*** | 1.9596 | 0.848371 | (0.00001, 5.77) |
| ***miR-135a*** | 0.4814 | 0.19272 | (0.00001, 1.44) |
| ***miR-135b*** | 0.2427 | 0.023229 | (0.00001, 0.75) |
| ***miR-146 a*** | 1.1486 | 0.927042 | (0.00001, 3.26) |
| ***miR-183*** | 0.7957 | 0.429944 | (0.00001, 2.34) |
| ***miR-223*** | 2.5515 | 0.190131 | (0.00001, 7.01) |
| ***miR-454*** | 0.4464 | 0.01808 | (0.00001, 1.29) |
| ***miR-24*** | 1.4921 | 0.919927 | (0.00001, 4.53) |
