# Supplementary material for: Circulating Serum miRNAs as Diagnostic Markers for Colorectal Cancer
Source: PLoS One. 2016 May 2;11(5):e0154130. doi: 10.1371/journal.pone.0154130 (PMC4852935; doi:10.1371/journal.pone.0154130)
Supplement: S3 Table — (DOC) [file pone.0154130.s003.doc]

**S3 Table: Differential expression of the studied miRNAs in CP group versus IBD group**

| **Gene Symbol** | **Fold change** | **p-value** | **95% CI** |
| --- | --- | --- | --- |
| ***miR-17*** | 2.1234 | 0.901303 | (0.00001, 6.95) |
| ***miR-18a*** | 3.8047 | 0.243132 | (0.00001, 10.42) |
| ***miR-19a*** | 2.8662 | 0.738294 | (0.00001, 8.88) |
| ***miR-20a*** | 0.5326 | 0.762003 | (0.00001, 1.37) |
| ***miR-21*** | 2.451 | 0.459156 | (0.00001, 7.93) |
| ***miR-92a*** | 1.0627 | 0.509767 | (0.00001, 2.72) |
| ***miR-183*** | 1.6895 | 0.590795 | (0.00001, 4.77) |
| ***miR-19b*** | 0.1219 | 0.619357 | (0.00001, 0.37) |
| ***miR-135a*** | 0.0906 | 0.019167 | (0.00001, 0.29) |
| ***miR-135b*** | 0.4345 | 0.018437 | (0.00001, 1.19) |
| ***miR-146 a*** | 1.1377 | 0.084358 | (0.00001, 3.48) |
| ***miR-223*** | 0.9802 | 0.375665 | (0.00001, 2.55) |
| ***miR-454*** | 0.4008 | 0.131821 | (0.00001, 1.24) |
| ***miR-24*** | 0.8435 | 0.192247 | (0.00001, 2.44) |
