# Supplementary material for: Circulating Serum miRNAs as Diagnostic Markers for Colorectal Cancer
Source: PLoS One. 2016 May 2;11(5):e0154130. doi: 10.1371/journal.pone.0154130 (PMC4852935; doi:10.1371/journal.pone.0154130)
Supplement: S4 Table — (DOC) [file pone.0154130.s004.doc]

**S4 Table: Differential expression of the studied miRNAs in female CRC group versus control group "Validation Set"**

| **Gene Symbol** | **Fold change** | **p-value** | **95% CI** |
| --- | --- | --- | --- |
| ***miR-17*** | 1.7735 | 0.083694 | (0.00001, 3.65) |
| ***miR-18a*** | 2.8942 | 0.074039 | (0.00001, 6.48) |
| ***miR-19a*** | 2.8058 | 0.02128 | (0.00001, 6.04) |
| ***miR-19b*** | 4.0697 | 0.023238 | (0.00001, 8.81) |
| ***miR-20a*** | 2.1606 | 0.043941 | (0.09, 4.23) |
| ***miR-21*** | 1.0446 | 0.162504 | (0.00001, 2.93) |
| ***miR-92a*** | 0.9019 | 0.42763 | (0.00001, 2.46) |
| ***miR-135a*** | 2.7886 | 0.448186 | (0.00001, 7.21) |
| ***miR-135b*** | 1.74 | 0.378062 | (0.00001, 5.90) |
| ***miR-146 a*** | 1.0864 | 0.185239 | (0.00001, 3.08) |
| ***miR-183*** | 2.8391 | 0.119024 | (0.00001, 5.86) |
| ***miR-223*** | 1.7251 | 0.095073 | (0.00001, 7.46) |
| ***miR-454*** | 12.1701 | 0.125318 | (0.00001, 34.27) |
| ***miR-24*** | 11.6098 | 0.125698 | (0.00001, 30.03) |
