# Supplementary material for: Circulating Serum miRNAs as Diagnostic Markers for Colorectal Cancer
Source: PLoS One. 2016 May 2;11(5):e0154130. doi: 10.1371/journal.pone.0154130 (PMC4852935; doi:10.1371/journal.pone.0154130)
Supplement: S5 Table — (DOC) [file pone.0154130.s005.doc]

**S5 Table: Differential expression of the studied miRNAs in male CRC patients versus control group "Validation Set"**

| **Gene Symbol** | **Fold change** | **p-value** | **95% CI** |
| --- | --- | --- | --- |
| ***miR-17*** | 2.1054 | 0.032758 | (0.00001, 4.97) |
| ***miR-18a*** | 1.9675 | 0.096828 | (0.16, 3.77) |
| ***miR-19a*** | 1.9065 | 0.081936 | (0.00001, 5.05) |
| ***miR-19b*** | 1.4097 | 0.271069 | (0.00001, 3.37) |
| ***miR-20a*** | 1.6379 | 0.085968 | (0.00001, 4.08) |
| ***miR-21*** | 1.4343 | 0.336982 | (0.00001, 2.87) |
| ***miR-92a*** | 0.7709 | 0.703692 | (0.00001, 1.77) |
| ***miR-135a*** | 0.8935 | 0.481728 | (0.00001, 2.80) |
| ***miR-135b*** | 0.3038 | 0.408033 | (0.00001, 1.10) |
| ***miR-146 a*** | 1.666 | 0.085661 | (0.00001, 3.90) |
| ***miR-183*** | 1.3641 | 0.21711 | (0.00001, 4.06) |
| ***miR-223*** | 5.897 | 0.059058 | (0.00001, 20.86) |
| ***miR-454*** | 0.8376 | 0.45674 | (0.00001, 2.22) |
| ***miR-24*** | 1.8226 | 0.152026 | (0.00001, 4.45) |
